# Collateral Damage: Detrimental Effect of Antibiotics on the Development of Protective Immune Memory

**DOI:** 10.1128/mBio.01520-16

**Published:** 2016-12-20

**Authors:** Joseph M. Benoun, Jasmine C. Labuda, Stephen J. McSorley

**Affiliations:** Center for Comparative Medicine, Department of Anatomy, Physiology and Cell Biology, School of Veterinary Medicine, University of California, Davis, Davis, California, USA

## Abstract

Antibiotic intervention is an effective treatment strategy for many bacterial infections and liberates bacterial antigens and stimulatory products that can induce an inflammatory response. Despite the opportunity for bacterial killing to enhance the development of adaptive immunity, patients treated successfully with antibiotics can suffer from reinfection. Studies in mouse models of *Salmonella* and *Chlamydia* infection also demonstrate that early antibiotic intervention reduces host protective immunity to subsequent infection. This heightened susceptibility to reinfection correlates with poor development of Th1 and antibody responses in antibiotic-treated mice but can be overcome by delayed antibiotic intervention, thus suggesting a requirement for sustained T cell stimulation for protection. Although the contribution of memory T cell subsets is imperfectly understood in both of these infection models, a protective role for noncirculating memory cells is suggested by recent studies. Together, these data propose a model where antibiotic treatment specifically interrupts tissue-resident memory T cell formation. Greater understanding of the mechanistic basis of this phenomenon might suggest therapeutic interventions to restore a protective memory response in antibiotic-treated patients, thus reducing the incidence of reinfection.

## INTRODUCTION

Since the discovery of penicillin in 1928, antibiotics have been widely used to treat bacterial infections, and as a result, bacteria have rapidly developed antibiotic resistance (1, 2). The development of multidrug-resistant (MDR) bacteria is now a critical issue in modern medicine, with the concern that serious bacterial infections will reemerge in the 21st century in the absence of effective treatment options (3–6). Despite this important issue, antibiotics remain an effective treatment option for many common infectious diseases.

An adaptive immune response to infection is initiated by recognition of foreign protein antigens in the presence of local inflammation (7). The contextual inflammatory cues come from innate immune cells that encounter bacterial products, and these signals profoundly affect the subsequent adaptive immune response (8). This initial activation stage occurs within local lymph nodes and causes low-frequency naive T cells and B cells to produce an army of effector cells to eradicate a complex pathogen (9, 10). Effective antibiotic therapy will kill a large number of bacteria, thus liberating antigen for lymphocyte recognition and releasing bacterial products that can amplify local inflammatory responses. Thus, antibiotics have a direct effect on bacterial growth but also have the potential to enhance an ongoing pathogen-specific adaptive immune response. However, many studies have shown that antibiotic administration can paradoxically weaken immune memory, leaving a recovered host fully susceptible to reinfection with the same pathogen (11–13). The mechanistic basis for this detrimental effect of antibiotics on immune memory and protection is incompletely understood. A more detailed understanding of this phenomenon might allow the development of targeted strategies to encourage immune memory development and support long-lasting protection from reinfection. In this review, we will discuss this issue in the context of recent findings from mouse models of *Salmonella* and *Chlamydia* infection, since both models show a detrimental effect of antibiotics upon the development of immune memory.

## HUMAN *SALMONELLA* AND *CHLAMYDIA* INFECTIONS

*Salmonella* bacteria cause a variety of clinical diseases, depending on the bacterial serovar and the underlying susceptibility of the infected host (14, 15). In many low-income countries with limited infrastructure, *Salmonella enterica* serovars Typhi and Paratyphi are transferred via the fecal-oral route and can cause enteric fever (16). While enteric fever can be successfully treated using antibiotics, the prevalence of multidrug-resistant strains is increasingly an impediment to treatment in areas where it is endemic (13). The administration of ciprofloxacin (a fluoroquine derivative) for 7 to 14 days is often sufficient to ensure the recovery of infected patients, but this depends upon the local prevalence of MDR strains (13, 17). Interestingly, even when treatment is successful, a cohort of patients suffer relapsing disease or can be reinfected with different Salmonella Typhi or Paratyphi strains at a later date. Thus, the successful resolution of primary infection with antibiotics does not guarantee the acquisition of protective immunity to reinfection.

*Salmonella* are not the only intracellular bacteria for which a lack of secondary protection is observed following antibiotic treatment. *Chlamydia trachomatis* is an obligate intracellular bacterium that causes ocular and sexually transmitted infections worldwide (18). In the United States, *Chlamydia* causes over 1.4 million sexually transmitted infections annually, and the health care costs associated with these infections amount to $500 million every year (19, 20). Immunity to *Chlamydia* infection in asymptomatic women develops slowly, and 50% of women continue to shed bacteria for a year (21). Since persistent or recurrent infection is a major risk factor for pelvic inflammatory disease (22, 23), *Chlamydia* control programs were introduced to reduce the burden of disease. These “seek and treat” programs have not reduced the incidence of *Chlamydia* infection but have reduced the incidence of associated pathology (24–27). However, reinfection is often observed following successful antibiotic treatment (24, 28), indicating that protective memory responses fail to develop in antibiotic-treated patients. Indeed, it has been argued that antibiotic treatment is counterproductive to the generation of *Chlamydia* immunity, an idea that is often referred to as the “arrested immunity” hypothesis (12). Recent clinical data support this hypothesis, since women who spontaneously resolve *Chlamydia* infection have a lower incidence of reinfection than antibiotic-treated women (29). Furthermore, gamma interferon (IFN-γ)-producing *Chlamydia*-specific Th1 cells develop slowly and do not persist in the circulation of women after effective antibiotic treatment (30). Thus, the high reinfection rates found in large population studies could actually be a consequence of early intervention programs that seek to screen and treat *Chlamydia*-infected women (24, 31). Together, these studies suggest findings that parallel those from *Salmonella*-infected patients and indicate that protective immunity does not develop effectively following antibiotic treatment of *Chlamydia*.

## PROTECTIVE IMMUNITY TO INTRACELLULAR BACTERIA

Since antibiotic treatment appears to have a negative impact on host protective immunity to secondary bacterial infection, it is vitally important to determine the mechanism of this phenomenon. The basic cellular immune responses to *Salmonella* and *Chlamydia* infection have been elucidated in mouse models and share common features (32–34). As expected for intracellular bacteria, CD4 Th1 cells that express T-bet and produce IFN-γ are critical for bacterial clearance. Thus, mice lacking major histocompatibility complex (MHC) class II-restricted T cells, T-bet, or IFN-γ succumb to primary infection with attenuated *S. enterica* serovar Typhimurium, an infection that resolves naturally in wild-type mice (35, 36). In contrast, mice lacking MHC class I-restricted CD8 T cells or B cells display only minor deficiencies in clearing primary *Salmonella* infection (37–39). Similarly, mice lacking MHC class II-restricted CD4 T cells or IFN-γ have difficulty resolving primary *Chlamydia* infection (40, 41), and yet, CD8 T cells or B cells are not essential (40, 42, 43). Together, these data point to a major role for CD4 Th1 cells in primary clearance of both *Salmonella* and *Chlamydia* infections. However, despite the fact that *Salmonella* and *Chlamydia* replicate intracellularly, antibody responses can play an important additive role during secondary infection (38, 39, 44, 45). Thus, memory CD4 T cells and circulating antibody can both be involved in effective clearance of bacteria during secondary infection (32, 33).

Persisting memory T cells contain at least three distinct subsets, each displaying different functional capabilities and tissue-homing potential (46, 47). Central memory T cells (TCM) recirculate between the blood and lymph fluid and have low immediate effector potential, similar to naive T cells. In contrast, effector memory T cells (TEM) display high immediate effector potential and can recirculate between blood and nonlymphoid tissues, anatomical locations where they are likely to encounter secondary bacterial infection. Finally, a population of resident memory T cells (TRM) remains within nonlymphoid tissues and has high immediate effector potential. This heterogeneity in T cell memory is important, since some infections show a greater reliance on tissue-resident versus circulating memory cells for pathogen clearance (48). While the protective contribution of distinct T cell memory subsets has not been fully explored in *Salmonella* infection models, recent data demonstrate that TRM CD4 T cells are critically important for immunity to *Chlamydia* infection (49). Thus, deficiency in secondary protective immunity to *Salmonella* and *Chlamydia* infection seems likely to involve CD4 T cell memory and may reflect an alteration in generating a specific protective subset.

## ANTIBIOTIC CLEARANCE OF *SALMONELLA* IN THE MOUSE MODEL

Two different mouse models are commonly used to investigate the immune response to *Salmonella* infection (50). The first model involves infecting genetically resistant mice with virulent *Salmonella* Typhimurium, thus allowing detailed study of innate and adaptive immune responses during the natural resolution of *Salmonella* infection (51). The alternative approach is to infect genetically susceptible mice with attenuated *Salmonella* strains, again allowing basic analysis of immune responses to primary bacterial infection (10). The obvious caveat to this second model is that the bacteria used are not fully virulent; however, the basic mechanism of primary clearance appears similar in both models. As noted above, protective immunity to secondary infection requires the cooperation of CD4 T cells and *Salmonella*-specific antibody responses. Importantly, in the genetically resistant model, protective immunity to reinfection can be transferred by antibody alone (52, 53), making this model less useful for examining CD4 T cell memory. Since humans require MHC class II-restricted T cell responses for efficient resolution of *Salmonella* infection (54), the susceptible mouse model is often used when studying the protective role of CD4 T cells against secondary infection.

Since many human typhoid infections are resolved by antibiotic treatment, our laboratory previously developed a mouse model in which susceptible mice were challenged with highly virulent *Salmonella* bacteria and antibiotics used to resolve primary infection. This proved more difficult than expected, and a full 5 weeks of enrofloxacin administration was required for C57BL/6 mice to completely resolve a primary infection with *Salmonella* Typhimurium (55). Live imaging experiments demonstrated that while viable bacteria were cleared from the spleen, liver, and bone marrow within 72 h of antibiotic treatment, a small population of bacteria persisted in mesenteric lymph node (MLN) phagocytes for several weeks after treatment (56). Thus, the removal of antibiotics before this population was eradicated allowed the outgrowth of bacteria and resumption of clinical disease. Indeed, relapse of primary infection was previously observed after treatment of murine infection with ampicillin and is also common to human salmonellosis (16, 57). Importantly, this relapsing disease does not require the development of antibiotic resistance, and these late-outgrowth bacteria remain susceptible to antibiotics (55). Similarly, in the resistant mouse model, recovery from primary infection is associated with continued bacterial shedding, indicating a chronic infection (58). Although persisting infection in both models is often localized to the MLNs (56, 58), surgical removal of the MLNs actually increased relapsing disease in antibiotic-treated mice (56), suggesting that the true site of bacterial persistence is more likely to be upstream from MLN afferent lymph drainage. *Salmonella* persistence has been correlated with the ability of some bacteria to enter a dormant state in which they are largely refractory to the effect of antibiotics (59, 60). However, recent studies have shown that outgrowing bacteria actually derive from bacteria with an intermediate growth phenotype that remain partially sensitive to antibiotic treatment (61). Thus, *Salmonella* bacteria display the ability to persist in the face of antibiotic treatment and in mice genetically predisposed to resolve primary infection. Given the wide availability of bacterial antigens for recognition by the host innate and adaptive immune system in these models, it is perplexing that relapsing infection can still occur many weeks after primary infection. The inability of the host immune system to effectively clear persistent or relapsing *Salmonella* infection might be due to effective immune evasion strategies employed by the bacteria (62–64).

**FIG 1:**
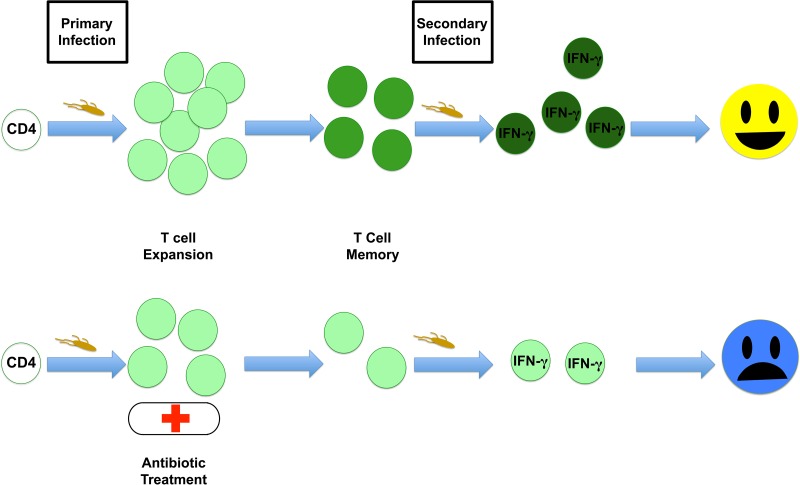
Antibiotic treatment reduces the development of T cell memory. Primary bacterial infection initiates the expansion of T cells that aid in clearance of the bacteria, leaving a pool of memory T cells behind. Upon secondary infection, these memory cells have acquired effector potential and can eliminate bacteria rapidly. Antibiotic treatment of primary infection truncates T cell expansion and limits memory cell development, preventing an effective response to secondary infection.

Susceptible mice treated for at least 5 weeks with enrofloxacin were able to fully resolve primary *Salmonella* infection, and these mice did not suffer from relapsing disease (55). This model therefore allowed a comparison of secondary protective immunity following antibiotic resolution of infection and naturally resolved primary infection with attenuated bacteria. While mice administered attenuated bacteria generated robust protective immunity to secondary infection, antibiotic-treated mice remained susceptible to *Salmonella* infection, although they survived longer than naive mice (55). These data point to an underlying deficiency in the acquisition of protective memory following antibiotic treatment that is similar to the susceptibility to reinfection observed in human disease (16). The weak protective immunity that was evident in antibiotic-treated mice required MHC class II-restricted T cells, B cells, and IFN-γ (55). When adaptive immune responses were examined in antibiotic-treated mice, it was noted that antibody responses were slightly depressed and the frequency of IFN-γ-producing cells was significantly reduced (55, 56). In recent experiments using MHC class II tetramers to track endogenous CD4 T cell responses to *Salmonella*, our laboratory has found that antibiotic treatment reduces the overall size of the *Salmonella*-specific CD4 memory pool (J. M. Benoun and S. J. McSorley, unpublished data). Thus, appropriate protective mechanisms appear to be engaged in antibiotic-treated mice, but the overall response is reduced, preventing a robust response to secondary infection (Fig. 1).

Previous studies have shown that CD4 clonal expansion and memory formation require sustained antigen presentation over several days (65, 66). Although antibiotic treatment liberates antigens from dead bacteria, it seems likely that this period of antigen presentation could be short, thus adversely impacting CD4 memory. Indeed, when antigen presentation is prolonged by delaying antibiotic intervention, this allows robust protective responses to emerge (56). This increased protection also coincides with a gradual increase in *Salmonella*-specific Th1 responses (56), suggesting that a prolonged period of antigen stimulation allows the recovery of memory development. Indeed, full recovery of Th1 responses required 14 days of exposure to live bacteria prior to antibiotic intervention (56). Thus, a major factor driving the detrimental impact of antibiotics upon protective memory responses is rapid elimination of bacterial antigens before memory CD4 T cells fully develop. Since CD8 T cells are thought to be less dependent on sustained antigen presentation (65–68), infections that rely on CD8 T cells for pathogen clearance might show less of a detrimental effect of antibiotics upon memory development. However, although initial CD8 responses occur normally with only a short period of antigen presentation, CD8 memory responses are still dependent to some extent upon prolonged antigen stimulation (69). In addition to sustained antigen stimulation, CD4 T cells also require costimulatory signals and local cytokines to develop appropriate functional responses (7). The availability of these costimulatory signals and cytokines is also likely to diminish as antibiotics rapidly resolve a primary bacterial infection. It will therefore be important to define precisely which of these signals is lacking in antibiotic-treated mice, since it may be possible to deliver these signals therapeutically during the period of antibiotic treatment.

## ANTIBIOTIC CLEARANCE OF *CHLAMYDIA* IN THE MOUSE MODEL

Investigators studying immunity to *Chlamydia* make use of two complementary mouse models (32, 70). In the first model, investigators use *Chlamydia trachomatis* to infect in-bred mouse strains, which has the obvious advantage of using the human pathogen directly in these model studies. However, *Chlamydia trachomatis* is not a natural pathogen of mice, and many laboratories therefore choose to examine immunity to *Chlamydia muridarum*, which causes an ascending reproductive tract infection following vaginal inoculation. *Chlamydia* bacteria have the ability to fuse and form noninfectious aberrant bodies under antibiotic pressure, and this form of the pathogen is known to be refractory to antibiotic killing (71, 72). There is also evidence that *Chlamydia* can persist in the intestine during an immune response or antibiotic treatment that can effectively clear bacteria from the reproductive tract (73, 74). Thus, *Chlamydia* and *Salmonella* each have the ability to persist in the face of antibiotic treatment, and for both these organisms, this may involve low-level chronic infection of intestinal tissues.

Secondary protective immunity has been examined following antibiotic clearance of *Chlamydia* infection in the mouse model. As with the *Salmonella* mouse model, successful treatment of *Chlamydia* infection in mice reduced protective immunity to subsequent secondary challenge compared to the protective immunity in mice that resolved primary infection naturally (75). Similar to *Salmonella* infection, the mechanistic basis of this arrested immunity is largely unknown but can be mitigated by delaying the start of antibiotic treatment (75), presumably because this allows more time for CD4 T cell memory to develop. Interestingly, administering a suboptimal dose of antibiotic creates a self-limiting subclinical infection, and these mice develop stronger adaptive responses and robust protective immunity as a result (76). The detrimental effect of antibiotic intervention in the *Chlamydia* model also correlates with lower *Chlamydia*-specific antibody responses and a reduced ability of splenocytes to produce IFN-γ in response to *Chlamydia* antigens (75). Thus, similar to the *Salmonella* model, rapid bacterial clearance using antibiotics is associated with the development of a dysfunctional memory response.

While CD4 T cells are essential for immunity to genital *C. muridarum* infection, the specific contribution of each TCM, TEM, and TRM subset has yet to be firmly established. Importantly, noncirculating tissue-resident memory (TRM) T cells have been identified as an essential component of protection at mucosal surfaces (77). Cells of this population typically have rapid effector capability, adopt tissue-specific differentiation patterns, and can initiate rapid innate immune responses to secondary infection (77–79). However, given the tissue-restricted localization of TRM T cells and the difficulty quantifying this population by flow cytometry, a comparative analysis of these subsets in the *Chlamydia* model is not trivial to complete (80). Recent studies have shown that repeated use of antibiotics to control a primary *Chlamydia* infection induces effector memory CD4 T cells in local draining lymph nodes (81), but the relationship between this population and effective protective immunity has not been established. As noted above, data from parabiosis experiments have shown that nonrecirculating memory cells are critical for protective immunity using a prototype *Chlamydia* vaccine (49). Thus, noncirculating TRM T cells would seem to be an integral component of protective immunity to secondary infection with *Chlamydia* (34). Therefore, it seems likely that antibiotic treatment during Chlamydia infection will adversely affect the development of *Chlamydia*-specific TRM within the reproductive tract, but this issue has yet to be examined experimentally.

## CONCLUSION

A number of experiments from *Salmonella* and *Chlamydia* infection models suggest that early antibiotic intervention impedes the development of effective protective memory, which is largely mediated by Th1 CD4 cells. The duration of antigen presentation and inflammatory stimulation are known to be key variables in the generation of CD4 T cell memory, and both of these variables are likely to be adversely affected by antibiotic administration. It is not yet clear whether this deficiency in CD4 T cell responses could be overcome by administering additional antigen or adjuvants during the period of antibiotic administration. Using the *Salmonella* infection model, we administered purified bacterial flagellin to mice being treated with antibiotic and detected the recovery of robust protective immunity to secondary infection (55). This experiment suggests that it may be possible to support the development of an effective memory lymphocyte population during antibiotic administration and that this can ultimately be of considerable benefit to the host. However, given the role of bacterial flagellin as a CD4 T cell target antigen and an inducer of inflammatory responses (82, 83), it still remains unclear whether providing antigen or adjuvant is the most effective strategy to recover protective memory responses. Future experiments will focus on whether the immune responses that are boosted in this context come from circulating or noncirculating memory cells. Greater understanding of the mechanisms of impaired adaptive immune responses after antibiotic treatment may allow simple and effective therapeutic strategies that could be easily translated to protect against repeated bacterial infections.
